# Exploring the efficacy of baricitinib in treating alopecia areata after failed Janus kinase inhibitor therapy

**DOI:** 10.1016/j.jdcr.2023.11.001

**Published:** 2023-11-14

**Authors:** Olivia Katamanin, Peter Yi Ch’en, Eingun James Song

**Affiliations:** a Chicago Medical School, Rosalind Franklin University, North Chicago, Illinois; b Albert Einstein College of Medicine, Bronx, New York; c Frontier Dermatology, Mill Creek, Washington

**Keywords:** alopecia areata, baricitinib, case series, JAK inhibitors, prior JAK inhibitor failure, treatment

## Introduction

Severe alopecia areata (AA), defined by hair loss ≥50% of the scalp, was traditionally treated with various off-label therapies including systemic steroids, immunosuppressants, and contact sensitization.^1^ The Food and Drug Administration approval of baricitinib in June 2022, a selective Janus kinase (JAK) 1/2 inhibitor, marked the first on-label treatment for AA.^2^ Before baricitinib, several case reports of oral tofacitinib, a JAK1/3 inhibitor, and oral ruxolitinib, a JAK1/2 inhibitor, demonstrated the potential to treat AA by targeting the JAK/STAT pathway.^3^ Yet, there were still a significant number of nonresponders to these agents which poses the question of whether alternative JAK inhibitors with different selectivity may still be beneficial in these patients. Because prior JAK inhibitor use was an exclusion criteria in the BRAVE AA-1 and BRAVE AA-2 trials, there is currently limited data to support the use of baricitinib in prior JAK inhibitor failures. Herein, we report our experience in 3 patients with severe AA that have failed a prior JAK inhibitor. Table I details the baseline demographics and treatment outcomes for each patient. The Severity of Alopecia Tool (SALT) was used to describe the extent of scalp-hair loss in patients.

**Table I:** Demographics and treatment outcomes

Variable	Case 1	Case 2	Case 3
Age (y) at initial visit, sex	70/F	56/F	73/F
PMH	Asthma, hyperlipidemia, hypothyroidism	Hypothyroidism	Hypothyroidism, AD, hypertension, DM2
SALT baseline	53 (AT previously)	AU	AU
Duration of disease	>10 y	3	1
Prior Rx	Prednisone, cyclosporine, methotrexate, intralesional kenalog injections, fexofenadine, ezetimibe/simvastatin, diphenylcyclopropenone	Prednisone, methotrexate, tofacitinib, low dose oral minoxidil	Prednisone, cyclosporine, methotrexate, intralesional kenalog injections, dupilumab, low dose oral minoxidil, tofacitinib
Baricitinib dose (daily)	4 mg	4 mg	4 mg
SALT response	20	40	5
Clin-RO eyebrow/lashes	0	0	0
Time off of previous JAKi before starting baricitinib	12 months off of upadacitinib	3 months off of tofacitinib	6 months off of upadacitinib 24 months off of tofacitinib
Duration of baricitinib (mo)	6	9	8

*AD*, Atopic dermatitis; *AT*, alopecia totalis; *AU*, alopecia universalis; *Clin-RO*, clinician reported outcome; *DM2*, type 2 diabetes mellitus; *PMH*, past medical history; *Rx*, treatment; *SALT*, Severity of Alopecia Tool.

## Case 1

**Fig 1:**
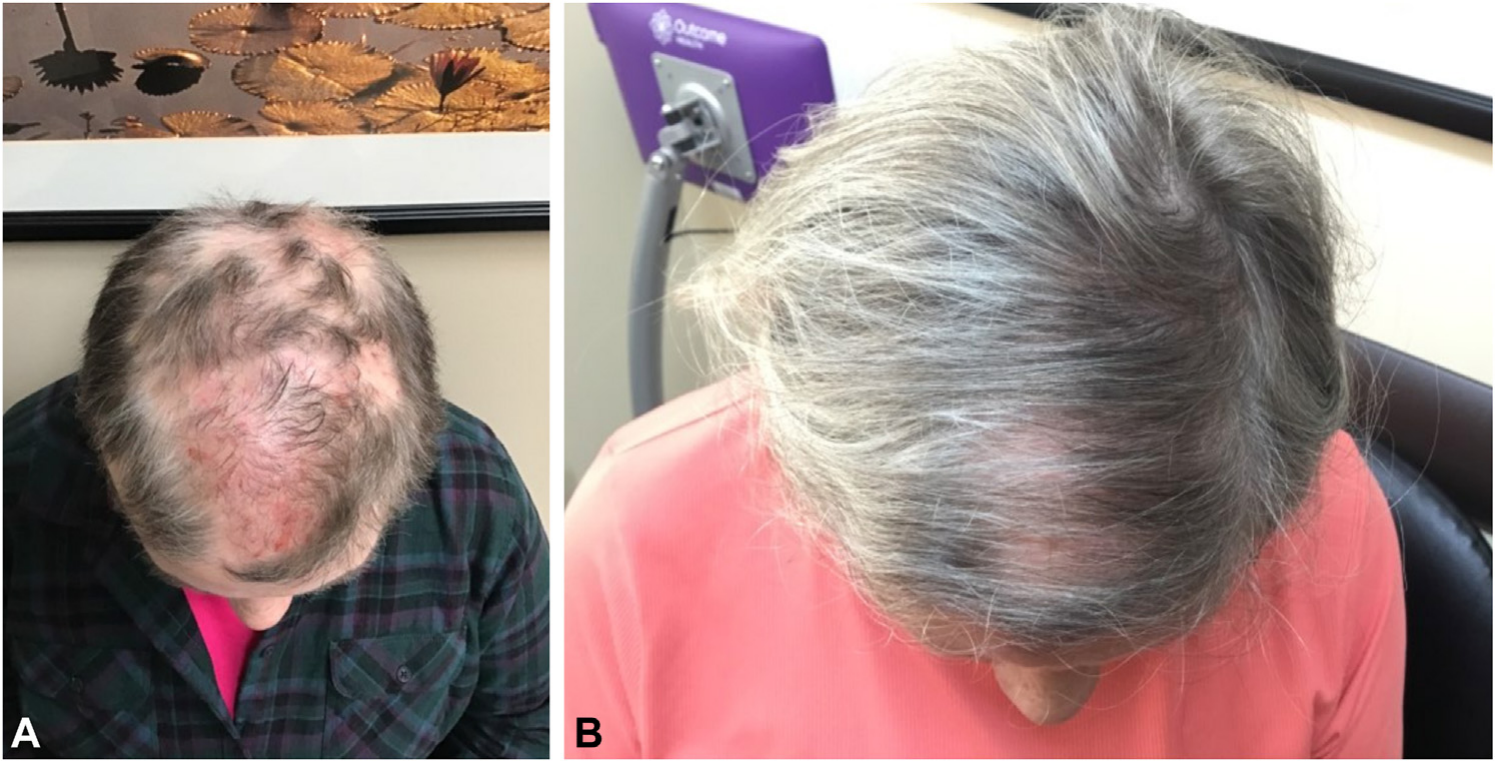
**A,** Pretreatment image of case 1 showing multiple areas of nonscarring alopecia after failing upadacitinib. **B,** Posttreatment image of case 1 showing marked improvement after 6 months.

A 70-year-old female presented to our dermatology clinic with a SALT baseline of 53 and disease duration of more than 10 years (Fig 1, *A*). Her affected areas included her frontoparietal scalp and occiput. Her past medical history was significant for asthma, hyperlipidemia, and hypothyroidism. She was previously treated with off-label upadacitinib 30 mg daily, a selective JAK1 inhibitor, for 6 months without any appreciable hair growth. Other treatments included prednisone, cyclosporine, methotrexate, intralesional kenalog injections, fexofenadine, ezetimibe/simvastatin, and diphenylcyclopropenone. She had achieved partial response to methotrexate 22.5 mg/week for years but was never able to achieve SALT 20. The patient was prescribed 4 mg of baricitinib daily for a 6 month duration in which she achieved SALT 20 (Fig 1, *B*).

## Case 2

**Fig 2:**
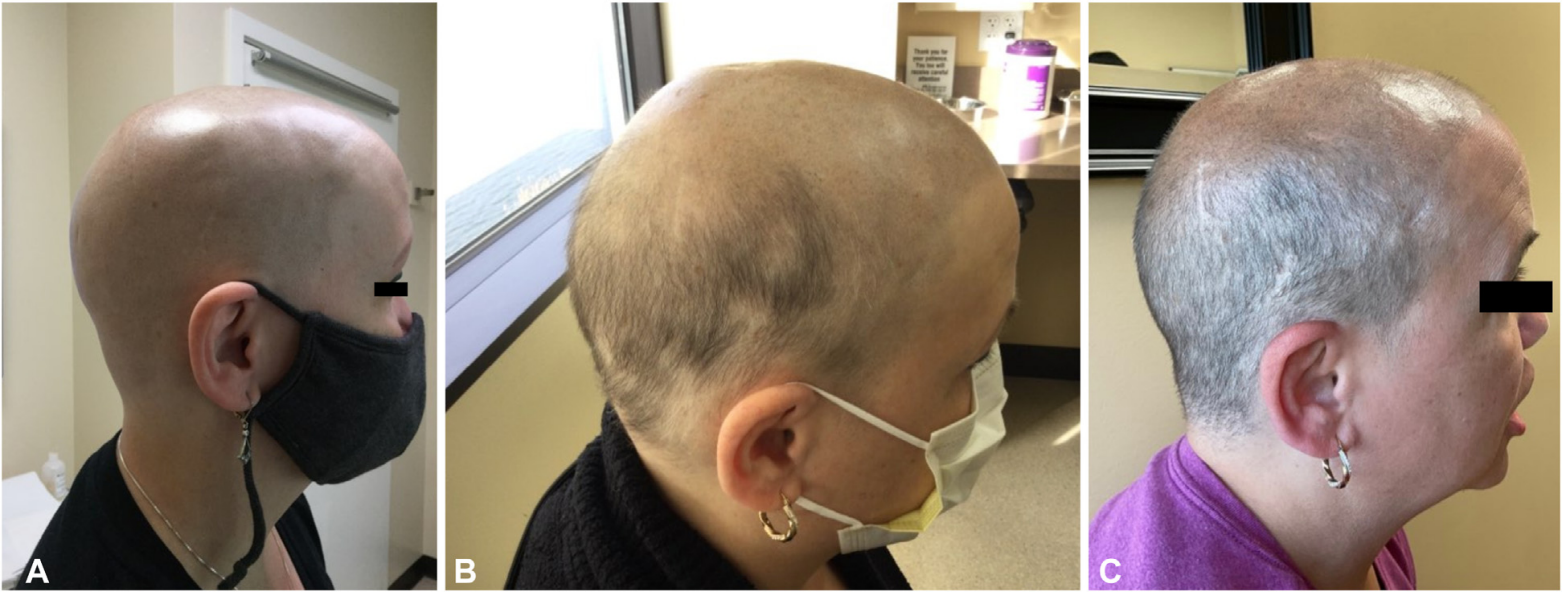
**A,** Pretreatment image of case 2 while on tofacitinib 11 mg extended release. **B,** Posttreatment image of case 2 showing slight improvement after 5 months of baricitinib 4 mg every day. **C,** Posttreatment image of case 2 demonstrating further improvement after 9 months of baricitinib 4 mg every day.

A 56-year-old female presented to our dermatology clinic with alopecia universalis with an ongoing disease duration of 3 years. Her past medical history was significant for hypothyroidism. She was treated with tofacitinib 11 mg extended-release which resulted in her eyebrows and eyelashes growing back but no scalp-hair growth after 1.5 years of treatment (Fig 2, *A*). Prior treatments included prednisone, methotrexate, and low dose oral minoxidil. The patient was prescribed 4 mg of baricitinib daily for a 9 month duration in which her SALT decreased to 40. While the patient’s temporal and occipital scalp fully grew, her vertex was still bald (Fig 2, *B* and *C*).

## Case 3

**Fig 3:**
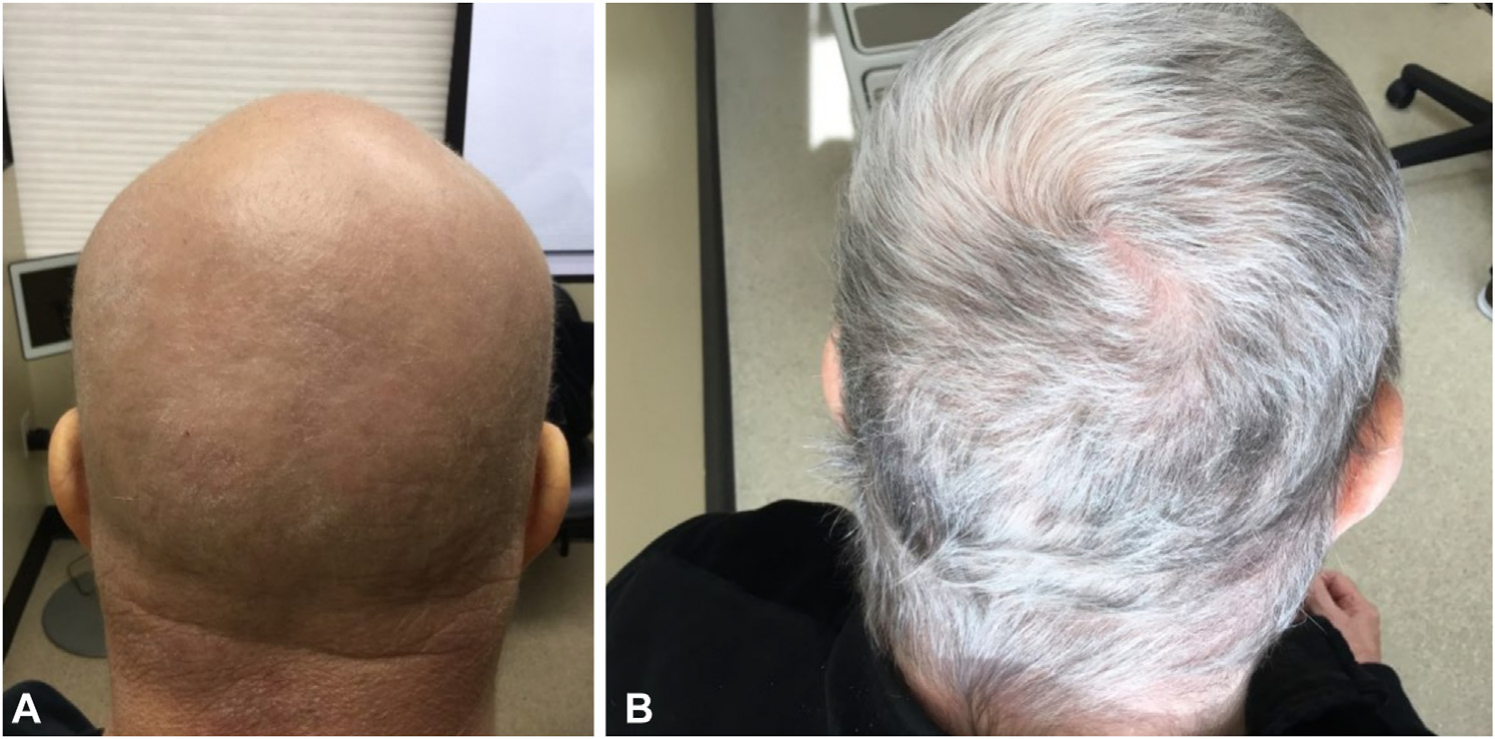
**A,** Pretreatment image of case 3 after failing upadacitinib. **B,** Posttreatment image of case 3 showing marked improvement after 8 months of baricitinib 4 mg every day.

A 73-year-old female presented to our dermatology clinic with alopecia universalis with an ongoing disease duration of 1 year. Her past medical history was significant for hypothyroidism, atopic dermatitis (AD), hypertension, and type 2 diabetes mellitus. She was previously treated with prednisone, cyclosporine, methotrexate, and intralesional kenalog injections. She actually showed near-complete hair regrowth with dupilumab but developed severe conjunctivitis which was unresponsive to various therapies from ophthalmology which prompted her to temporarily discontinue. Shortly after stopping dupilumab, the patient lost all of her hair and was never able to recapture after restarting dupilumab. Patient was then started on tofacitinib 11 mg extended release but did not show any appreciable hair growth at 6 months and was unable to continue due to loss of foundation support from the drug manufacturer. Because the patient also had concomitant moderate-to-severe AD, she was started on upadacitinib 15 mg daily with up titration to 30 mg at 3 months. While her AD completely cleared, she achieved just partial hair regrowth after 6 months of therapy and subsequently relapsed to a SALT 100 (Fig 3, *A*). Patient was subsequently started on baricitinib 4 mg daily and by 8 months had achieved SALT 5 (Fig 3, *B*). However, her AD has worsened and is being managed with topical corticosteroids.

## Discussion

Significant advancements have been made in the treatment of severe AA since June 2022, with now 2 Food and Drug Administration-approved medications in baricitinib, a JAK1/2 inhibitor, and ritlecitinib, a JAK3/TEC inhibitor. Both treatments have demonstrated the potential for near-complete hair growth in a significant number of patients with an acceptable safety profile.^2^^,^^4^^,^^5^ Still, >60% of patients still did not reach the primary end point of SALT 20 with both medications, demonstrating the need for additional therapies. While it can be useful to think about medications in a certain class together, differences in JAK selectivity can confer efficacy and safety advantages.^6^ In both rheumatoid arthritis and psoriatic arthritis where JAK inhibitors are also Food and Drug Administration-approved treatments, it has been shown that certain patients who failed a prior JAK inhibitor can benefit from switching to another JAK inhibitor.^7^ Likewise, we have shown in a small number of patients that prior JAK inhibitor failure should not preclude the use of baricitinib.

Pharmacologic inhibitory differences of the various JAK isoforms may lead to greater inhibition of relevant AA cytokines, namely interleukin-15 and interferon-gamma, and may explain differences in clinical efficacy. As first generation JAK inhibitors, tofacitinib and baricitinib will inhibit a broader range of cytokines than upadacitinib which may be advantageous in a heterogenous disease such as AA.^8^ Furthermore, because JAK inhibition and selectivity is dose-dependent, it is possible that using a higher dose of tofacitinib and upadacitinib would have yielded better results. In addition, differences in drug half-life, tissue penetration and distribution, individual variations in single nucleotide polymorphisms affecting STAT isoforms, and differential expression of JAK expression at sites of inflammation may explain differences in treatment response with a particular JAK inhibitor.^9^

Our case series also brings to the light the importance of considering comorbidities. While baricitinib is approved in Japan and European Union for moderate-to-severe AD, the efficacy was considerably lower than what was seen in the pivotal trials.^10^ Not unsurprisingly, patient 3 had a flare in her AD after switching to baricitinib.

The limitations of this case series include a small sample size and a fairly short follow-up period. Although all 3 patients started with the 4 mg dose of baricitinib, it is unclear if the 2 mg dose would have been sufficient. Future research on identifying patient characteristics that may identify a more favorable response to a particular JAK inhibitor is needed.

## Conflicts of interest

None disclosed.
